# Quality of information on fertility clinic websites accredited by the Latin American Network of Assisted Reproduction

**DOI:** 10.5935/1518-0557.20240074

**Published:** 2025

**Authors:** Giselle Ferreira de Souza, Eduarda Maia Lima, Ana Clara Muniz Tavares, Camila Alves Rocha, Larissa Cruz de Souza, Júlia Machado Luz Simões, Nicole Oliveira de Araújo, Maria Yzadora Moura Martins, Marcelo Borges Cavalcante

**Affiliations:** 1 Medical Course, Universidade de Fortaleza (UNIFOR), Fortaleza, CE, 60.811-905, Brazil; 2 Graduate Program in Medical Sciences, Universidade de Fortaleza (UNIFOR), Fortaleza, CE, 60.811-905, Brazil; 3 CONCEPTUS - Reproductive Medicine, Fortaleza, CE, 60.170-240, Brazil

**Keywords:** assisted reproductive technology, *in vitro* fertilization, internet, website, quality, advertising

## Abstract

**Objective:**

To access to reproductive health information on the Internet helps patients understand their infertility journey and make decisions about their treatment. This study aimed to evaluate the quality of fertility clinic websites accredited by the Latin American Network for Assisted Reproduction (REDLARA) using the QUality Evaluation Scoring Tool (QUEST).

**Methods:**

This observational, cross-sectional, and online study evaluated the clinic websites registered as accredited centers on the REDLARA website. The QUEST was used for the quality assessment of the websites. Data were collected from the available websites of all accredited fertility clinics between September 2023 and January 2024.

**Results:**

A total of 173 websites from fertility clinics accredited by REDLARA were evaluated, and 152 (87.8%) clinics had functioning websites. The majority of analyzed websites were from Brazilian fertility clinics (n=58; 38.1%), followed by Mexican (n=23; 15.1%) and Argentine (n=21; 13.8%). No indication of authorship or username was observed on most websites. Some form of support for the patient-physician relationship was reported by 86.8% of websites. The mean (standard deviation±SD) of the total score obtained by all fertility clinics was 12.73±4.7 (range: 1-26). Brazil had the highest total score (mean±SD=16.03±4.6), whereas Peru had the lowest (6.42±1.7). Statistical analysis revealed a difference in the quality of websites among Latin American countries.

**Conclusions:**

The health information disseminated by fertility clinic websites in Latin America is of poor quality. Therefore, REDLARA should implement rules for building good-quality websites.

## INTRODUCTION

The Latin American Network for Assisted Reproduction (REDLARA) is an institution that brings together private and public fertility clinics for scientific and educational purposes. Established in 1995, the main objectives of REDLARA are as follows: 1) to promote continuing education, 2) to encourage scientific updating, and 3) to compile, analyze, and publish treatment outcomes performed by accredited centers through annual data registration. REDLARA began its activities with 50 associated fertility clinics; however, since the beginning of its accreditation in 1997, it currently registers almost 200 centers, including Argentina, Brazil, Chile, Colombia, Costa Rica, Ecuador, El Salvador, Guatemala, Mexico, Panama, Peru, Dominican Republic, Uruguay, and Venezuela. Currently, REDLARA has a digital platform (www.redlara.com) with all information about the organization’s history and objectives and access to information from all accredited fertility clinics (REDLARA, 2024).

Globally, internet access is growing and influencing people’s behaviors and quality of life, becoming a vital part of everyday life (Kampmeijer *et al.*, 2016; Aggarwal *et al.*, 2020; Bujnowska-Fedak & Węgierek, 2020; Pryor *et al.*, 2022). In 2023, the number of Internet users worldwide currently connected to the World Wide Web was 5.3 billion people, comprising about two-thirds of the global population (Statista., 2024). Among the topics searched on the Internet, the frequent search for information about the health sector stands out (Finney Rutten *et al.*, 2019).

The information available on the Internet helps patients understand more about their health condition and creates autonomy to get involved in their well-being in a way that did not happen before the existence of the virtual world (Kushniruk, 2019; Bujnowska-Fedak & Węgierek, 2020; Campbell & Rudan, 2020). Furthermore, this process empowers patients to make informed decisions because access to up-to-date data about their medical condition, likely side effects, and available therapies opens up the possibility of evaluating risks and benefits of medical conduct, strengthening their involvement in the process, and increasing adherence to the chosen treatment (Fahy *et al.*, 2014; Ghazavi-Khorasgani *et al.*, 2018; McLean, 2023). However, as much as the Internet can mitigate misinformation and help patients learn about diseases, diagnoses, and treatments, it also has the potential to guide individuals incorrectly because not all information disseminated online is regulated and reliable (Ghazavi-Khorasgani *et al.*, 2018; Galiano *et al.*, 2021).

The patients’ well-being can be harmed by the dissemination of health information without strict control, in addition to interfering with decision-making about the management of health problems. Distorted information can result in dangerous choices, delaying the disease diagnosis or even choosing a therapy without proven benefits (Tonsaker *et al.*, 2014; Swire-Thompson & Lazer, 2020). Several studies have already observed the poor quality of information on websites about different health topics (Abusief *et al.*, 2007; Avraham *et al.*, 2014; Daraz *et al.*, 2019; Stern *et al.*, 2021).

In 2024, reproductive medicine celebrates the 46th anniversary of the first baby born through *in vitro* fertilization. Over the last four decades, diagnostic methods, assisted reproductive technology (ART), new ovarian stimulation protocols, and the addition of many add-ons to improve the live birth rate have greatly evolved (Johnson, 2019). However, a large number of interventions still lack robust scientific evidence to be integrated into clinical practice (ESHRE, 2023). Furthermore, knowledge about ART among the population is limited, as access to these treatments, in some countries, is restricted to some patients with better financial conditions (Daniluk & Koert, 2015; Oliveira *et al.*, 2021).

Fertility clinics worldwide publicize their work through their websites, containing information on the main topics in reproductive medicine, ART, treatment success rates, treatment costs, and clinical staff profile, in addition to informing the patient about the process of promoting healthy and safe conception. Studies have observed that the content published on fertility clinic websites in the United States of America (USA) and Brazil is heterogeneous and does not follow the American Society for Reproductive Medicine (ASRM) and Federal Council of Medicine (CFM) guidelines, respectively, not guaranteeing an adequate place for safe information for patients (Abusief *et al.*, 2007; Carneiro *et al.*, 2023). Therefore, the importance of websites adopting rigorous standards should be understood to allow patients to have access to reliable data to ensure that the clinics they choose to carry out their infertility treatment are transparent and provide homogeneous and accurate information (Huang *et al.*, 2005a; Carneiro *et al.*, 2023 ).

Given the importance of access to good-quality health information, several website quality assessment tools have already been proposed (Stvilia *et al.*, 2009; Robillard *et al.*, 2018). The American Medical Association (AMA) was one of the first associations to alert users about the importance of critically analyzing online content and defined guidelines to guarantee the quality of information and protect visitors’ data. These criteria include site ownership, ease of site navigation and viewing, user access, payment and privacy, sponsorships, editorial content quality, link access, and navigation (Winker *et al.*, 2000). Currently, no clear universal standard has been established for evaluating the quality of online health information; thus, many researchers and regulatory agencies have already proposed tools with different evaluation criteria, such as the QUality Evaluation Scoring Tool (QUEST) (Robillard *et al.*, 2018).

This study aimed to evaluate the quality of fertility clinic websites accredited by REDLARA using the QUEST tool.

## METHODS

### Design and participants

An observational, cross-sectional, online study was performed to evaluate the quality of fertility clinic websites in Latin America. The authors evaluated the clinic websites registered as accredited centers on the REDLARA website. The initial access to the accredited fertility clinics’ websites occurred through a redirection link on the REDLARA website (in the “Who We Are” tab). When accessing the website through redirection from the REDLARA website is impossible, the authors made a second attempt by searching for the name of the clinic on an online search platform (www.google.com).

### QUEST and data collection

**Figure 1:**
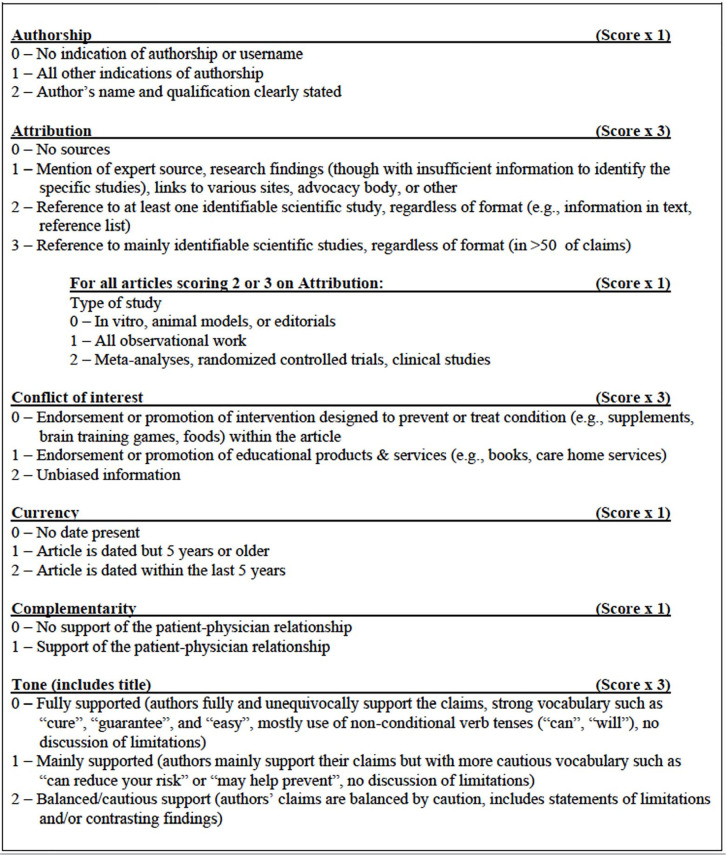
Description of the QUEST criteria. Scores in the individual sections are weighted and summed to generate a total score of up to 28. From: Robillard *et al*. The QUEST for quality online health information: validation of a short quantitative tool. BMC Med Inform Decis Mak. 2018;18:87.

The QUEST tool, a system used to evaluate and score the quality of a website based on various predefined criteria, was used to assess the quality of websites. This tool quantitatively measures six aspects of the quality of online health information: authorship, attribution, conflict of interest, currency, complementarity, and tone. The overall quality score can range from 0 to 28 (Figure 1).

The authorship of the website is attributed to the individual or legal entity responsible for its administration and management, not necessarily including medical professionals who work at the clinic. Companies responsible for creating and designing websites were not included in this item. The attributions refer to bibliographic sources of the texts present in each tab/section of the website. If any of these sources are scientific articles, the sale of products or courses, whether to doctors who are not part of the clinical team or to interested patients, was considered a conflict of interest. The provision of services relating to human reproduction procedures does not represent a conflict of interest. Regarding dating, the publication dates of the texts present in the website’s tabs were analyzed. Regarding the doctor-patient relationship, the inclusion criteria refer to tabs/sections, question forums, or inviting messages for communication with clinic professionals through mechanisms on the website itself, but any item that was directed to an external link was disregarded. Finally, intonation refers to any message related to the clinic’s services and their results in the couple’s fertilization process.

In pairs, the authors (ACMT, CAR, EML, GFS, JMLS, LCS, MYM, and NOA) collected data from available websites of all accredited fertility clinics between September 2023 and January 2024, which were subsequently validated by the coordinating author of the study (MBC).

### Data analysis and ethical aspects

Data were summarized in tables and graphs. Statistical analysis was performed using GraphPad Prism software version 10.2.0. Data were summarized as frequencies (n) and percentages (%) for categorical variables and means or medians (standard deviation [SD] or interquartile range [IQR]) for continuous and ordinal variables, respectively. Each QUEST item and the total score obtained by clinics in each country were evaluated individually. The total scores of fertility clinics in each country were compared. The oneway analysis of variance (ANOVA) was used to compare the differences between the groups. The *p*-value of <0.05 was considered significant. The data used did not require approval from the ethics committee, given the use of public advertising information, and our data did not disclose information that might allow the identification of clinics.

## RESULTS

**Figure 2:**
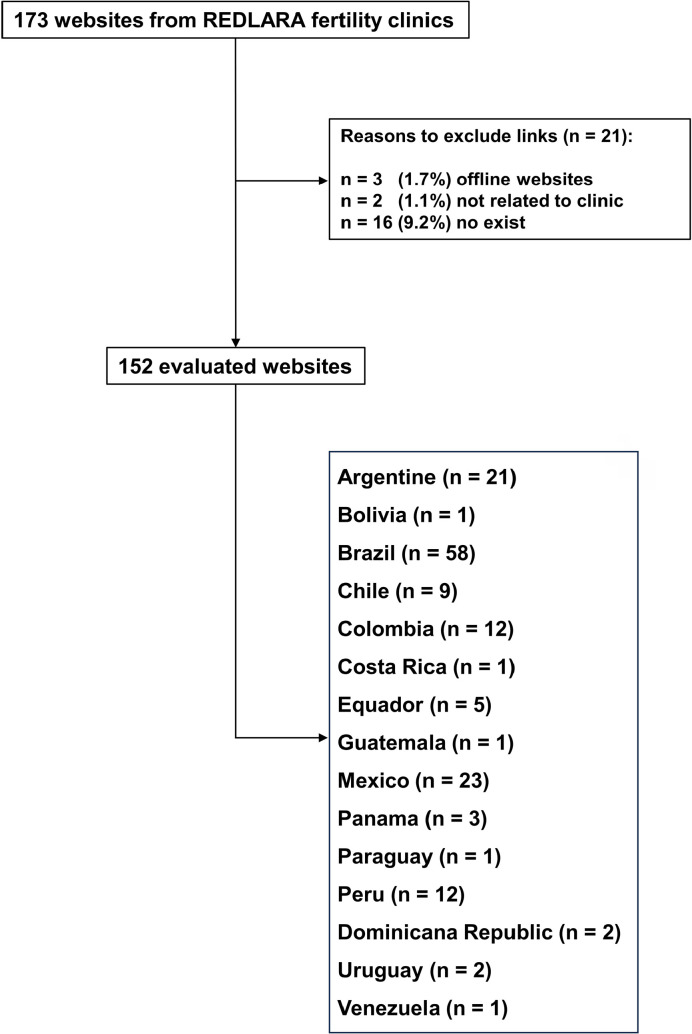
A total of websites from fertility clinics accredited by REDLARA.

A total of 173 websites from fertility clinics accredited by REDLARA were evaluated. A total of 152 (87.8%) clinics had functioning websites, three (1.7%) were offline, 2 (1.1%) provided links not compatible with the evaluated clinics, and 16 (9.2%) provided links to sites that did not exist (Figure 2). Table 1 summarizes the results of items evaluated by the QUEST tool. The majority of websites analyzed were from Brazilian fertility clinics (n=58; 38.1%), followed by Mexican (n=23; 15.1%), Argentine (n=21; 13.8%), Peruvian (n=12; 7.9%), Colombian (n=12; 7.9%), Chileans (n=9; 5.9%), Ecuadorians (n=5; 3.3%), Panamanians (n=3; 1.9%), Dominicans (n=2; 1.3%), Uruguayans (n=2; 1.3%), Venezuelan (n=1; 0.65%), Paraguayan (n=1; 0.65%), Guatemalan (n=1; 0.65%), Costa Rican (n=1; 0.65%), and Bolivian (n=1; 0.65%) (Figure 2).

**Table 1:** Items evaluated by Quality Evaluation Scoring Tool (QUEST) according to fertility clinics accredited by REDLARA in each country.

Country (n [%])	Authorship (Median [IQR])	Attribution (Median, [IQR])	Attribution 2 (Median, [IQR])	Conflict of interest (Median, [IQR])	Currency (Median, [IQR])	Complementarity (Median, [IQR])	Tone (Median, [IQR])	Total score (Mean ± SD)
Argentina (n=21 [13.8])	0 (0 - 0)	0 (0 - 3)	0 (0 - 1)	6 (6 - 6)	0 (0 - 2)	1(1-1)	3 (3 - 6)	12.73±4.7
Bolivia (n=l [0.6])^*^	0	0	-	6	2	0	0	8
Brazil (n=58 [38.1])	0 (0 - 0)	3 (0 - 6)	1 (0-_1.75)	6 (6 - 6)	2 (0 - 2)	1(1-1)	6 (3 - 6)	16.03±4.6
Chile (n=9 [5.9])	0 (0 - 0)	3 (0 - 3)	0 (0 - 0)	6 (6 - 6)	0 (0 - 2)	1 (0.5 - 1)	6 (3 - 6)	13.89±2.4
Colombia (n=12 [7.9])	0 (0 - 0)	0(0-_5.25)	-	6 (6 - 6)	2 (0 - 2)	1(1-1)	3 (3 - 3)	12.33±3.2
Costa Rica (n=l [0.6])^*^	0	3	-	6	0	1	3	13
Equador (n=5 [3.3])	0 (0 - 0)	0 (0 - 0)	-	6 (3 - 6)	0 (0 - 1)	1(1-1)	3 (3 - 6)	10.4±3.8
Guatemala (n=l [0.6])^*^	0	0	-	3	0	1	3	7
Mexico (n=23 [15.1])	0 (0 - 0)	0 (0 - 0)	-	6 (6 - 6)	0 (0 - 0)	1(1-1)	3 (3 - 6)	10.3±2.3
Panama (n=3 [1.9])	0 (0 - 0)	0 (0 - 3)	-	6 (3 - 6)	2 (0 - 2)	1(1-1)	3 (3 - 3)	11.33±3.2
Paraguay (n=l [0.6])^*^	0	0	-	6	0	0	0	6
Peru (n=12 [7.9])	0 (0 - 0)	0 (0 - 0)	1(1-1)	6 (3 - 6)	0 (0 - 0)	1(1-1)	0 (0 - 0)	6.42±1.7
Dominicana Republic (n=2[1.3])^*^	0 (0 - 0)	0 (0 - 0)	0 (0 - 0)	0 (0 - 0)	0 (0 - 0)	0 (0 - 0)	0 (0 - 0)	7 and 7
Uruguay (n=2 [1.3])^*^	0 (0 - 0)	0 (0 - 0)	0 (0 - 0)	0 (0 - 0)	0 (0 - 0)	0 (0 - 0)	0 (0 - 0)	7 and 7
Venezuela (n=l [1.3])^*^	0 (0 - 0)	0 (0 - 0)	0 (0 - 0)	0 (0 - 0)	0 (0 - 0)	0 (0 - 0)	0 (0 - 0)	6
Overall (n=152)	0 (0 - 0)	0 (0 - 3)	0 (0 - 1)	6 (6 - 6)	0 (0 - 2)	1(1-1)	3 (3 - 6)	12.73±4.7

IQR: interquartile range; SD: standard deviation.

* The total score values reported are the individual values of each fertility clinic evaluated.

**Figure 3:**
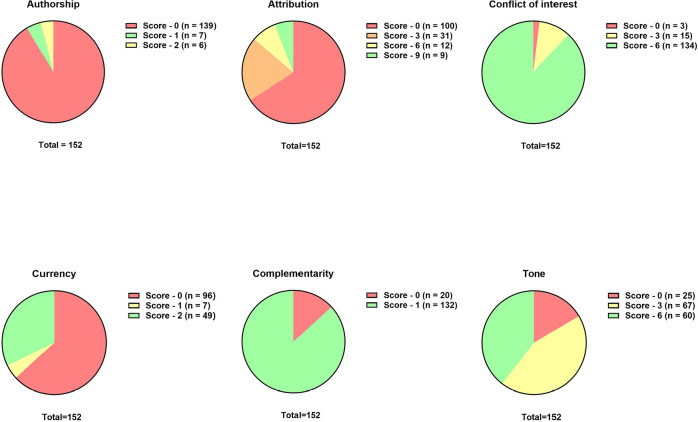
Frequency each item of the QUality Evaluation Scoring Tool.

Figure 3 summarizes the frequency each item of the QUEST tool was evaluated. No indication of authorship or username was observed on the vast majority of websites (n=139/152, 91%). The maximum score for the attribution item occurred in 5.9% of websites evaluated (n=9/152). A conflict of interest was observed in 11.8% of the websites (n=18/152). Only 31.6% (n=48/152) reported a website update date of <5 years apart. Some form of support for the patient-physician relationship was reported by the vast majority of websites (n=132/152, 86.8%). Regarding the intonation of information, 25 sites received a grade of 0 (16.44%), 67 received a grade of 3 (44.07%), and 60 received a grade of 6 (39.47%).

**Figure 4:**
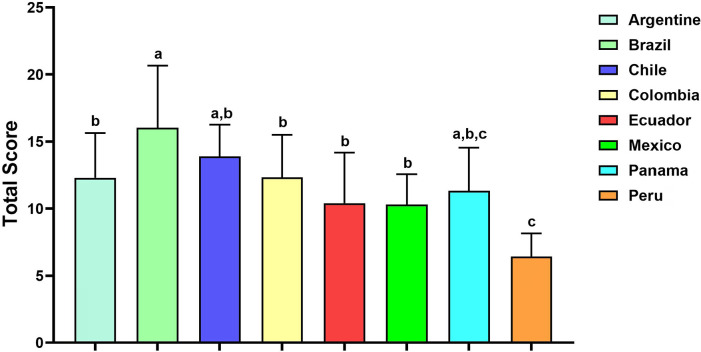
Total score of fertility clinics accredited by REDLARA per country. The data are graphed as mean±SD, one-way analysis of variance was used to compare the differences between the countries and that *p*<0.05 for statistical significance. The letters at the top of the bars (a, b, and c) mark significant differences of total score between the countries. Countries with the same letter, the difference between the means is not statistically significant. Countries with different letters, the total scores were significantly different.

The mean (±SD) of the total score obtained by all clinics was 12.73±4.7 (range:1-26). No website achieved the maximum total score for the QUEST tool. Brazil was the country with the highest total score (mean±SD=16.03±4.6), followed by Chile (13.89±2.4) and Argentine (12.73±4.7), whereas Peru had the lowest overall score (6.42±1.7). Statistical analysis using the ANOVA test revealed a difference in the quality of websites between Latin American countries (Figure 4).

## DISCUSSION

The Internet has become an accessible source of information for learning about health and can be an ally for early disease diagnosis, as well as greater understanding and patient adherence to the proposed treatment plan (Bujnowska-Fedak & Węgierek, 2020). However, several studies warn about the amount of inaccurate information on websites with health-related topics (Abusief *et al.*, 2007; Avraham *et al.*, 2014; Daraz *et al.*, 2019).

Studies from the early 2000s have already observed inconsistent information on American fertility clinic websites. The vast majority of fertility clinic websites did not comply with the ASRM guidelines (Huang *et al.*, 2005b; Abusief *et al.*, 2007). Other authors also observed low-quality information on social cryopreservation of oocytes provided by the websites of ASRM member clinics (Avraham *et al.*, 2014). Therefore, professional reproductive medicine organizations in the USA, including ASRM and the Society for Assisted Reproductive Technology (SART), have proposed guidelines to standardize reproductive health information on the Internet (American Society for Reproductive Medicine, 2021). An Australian study noted poor quality information about success rates provided on infertility clinic websites in Australia and New Zealand, even after intervention by the Australian Competition and Consumer Commission (Hammarberg *et al.*, 2018). Recently, a study reported that the vast majority of Brazilian fertility clinic websites did not follow ASRM and CFM recommendations. Only a third of websites published the name of a registered director. Moreover, 33% of clinics discussed success rates, only 11% used their success rates, and 4% reported success rates by age (Carneiro *et al.*, 2023).

Standardizing rules in developing websites for fertility clinics has been a topic of debate for decades. The search by patients for methods that can increase the success rates of infertility treatments and the offer of add-ons without scientific evidence on fertility clinic websites is a matter of debate among medical societies (Braga *et al.*, 2022). In the early 2000s, studies already warned about the poor quality of the websites of SART-affiliated clinics. Abusief *et al*. (2007) reported that a significant proportion of SART member fertility clinics do not follow ASRM/SART guidelines for advertising (Abusief *et al.*, 2007). In Brazil, the federal government has regulated medical marketing actions since 1942 (Brazil Decree-law no: 4113/1942). The CFM recently published new guidelines (CFM resolution 2336/2023) for the dissemination of medical information on websites, social media, and other printed or digital means of dissemination (CFM, 2023).

To our knowledge, this is the first study that evaluated the quality of fertility clinic websites accredited by REDLARA using the QUEST tool. The veracity level of the information disclosed by the evaluated websites is a limitation of the QUEST tool and consequently of this study. However, the study highlights the urgency of creating rules for the dissemination of medical information on fertility clinic websites in centers accredited by REDLARA. The evaluation of websites and communication channels of REDLARA member clinics must be part of the accreditation and reaccreditation process. The creation of a website quality seal is an alternative to encourage fertility clinics to follow guidelines established by REDLARA. Furthermore, the website’s certification by REDLARA ensures that patients have access to high-quality information.

In conclusion, the poor quality of health information disseminated by fertility clinic websites in Latin America can be observed. Fertility clinic websites must follow existing legislation in each country. Health regulatory agencies in Latin American countries should pay attention to the content of fertility clinic websites and seek to establish a collaboration with REDLARA, which can offer scientific consultancy.
